# Application of Spherical-Radial Cubature Bayesian Filtering and Smoothing in Bearings Only Passive Target Tracking

**DOI:** 10.3390/e21111088

**Published:** 2019-11-07

**Authors:** Wasiq Ali, Yaan Li, Zhe Chen, Muhammad Asif Zahoor Raja, Nauman Ahmed, Xiao Chen

**Affiliations:** 1 School of Marine Science and Technology, Northwestern Polytechnical University, Xi’an 710072, Shaanxi, China; chenzhe@mail.nwpu.edu.cn (Z.C.); nauman@mail.nwpu.edu.cn (N.A.); 2 Department of Electrical and Computer Engineering COMSATS University Islamabad, Attock Campus, Attock 43600, Pakistan; Muhammad.asif@ciit-attock.edu.pk; 3 School of Electronic Information and Artificial Intelligence, ShaanXi University of Science & Technology, Xi’an 710021, ShaanXi, China; chenxiao@sust.edu.cn

**Keywords:** Bayesian filtering, passive target tracking, spherical radial cubature Kalman filter, Gaussian measurement noise, Rauch–Tung–Striebel smoother

## Abstract

In this paper, an application of spherical radial cubature Bayesian filtering and smoothing algorithms is presented to solve a typical underwater bearings only passive target tracking problem effectively. Generally, passive target tracking problems in the ocean environment are represented with the state-space model having linear system dynamics merged with nonlinear passive measurements, and the system is analyzed with nonlinear filtering algorithms. In the present scheme, an application of spherical radial cubature Bayesian filtering and smoothing is efficiently investigated for accurate state estimation of a far-field moving target in complex ocean environments. The nonlinear model of a Kalman filter based on a Spherical Radial Cubature Kalman Filter (SRCKF) and discrete-time Kalman smoother known as a Spherical Radial Cubature Rauch–Tung–Striebel (SRCRTS) smoother are applied for tracking the semi-curved and curved trajectory of a moving object. The worth of spherical radial cubature Bayesian filtering and smoothing algorithms is validated by comparing with a conventional Unscented Kalman Filter (UKF) and an Unscented Rauch–Tung–Striebel (URTS) smoother. Performance analysis of these techniques is performed for white Gaussian measured noise variations, which is a significant factor in passive target tracking, while the Bearings Only Tracking (BOT) technology is used for modeling of a passive target tracking framework. Simulations based experiments are executed for obtaining least Root Mean Square Error (RMSE) among a true and estimated position of a moving target at every time instant in Cartesian coordinates. Numerical results endorsed the validation of SRCKF and SRCRTS smoothers with better convergence and accuracy rates than that of UKF and URTS for each scenario of passive target tracking problem.

## 1. Introduction

In the last two decades, a lot of nonlinear estimation approaches have been proposed from the research community for solving nonlinear state estimation problems [1]. These techniques are mainly applied to statistical nonlinear processes which are usually noise corrupted [2]. In this domain, Gaussian postulation based nonlinear filters have been thoroughly investigated due to their robustness and effectiveness in many real-life engineering applications [3,4,5]. The famous Bayesian filtering approach provides an effective solution to nonlinear state approximation problems, which identify the subsequent allocation of coordinates for the real-time motion parameters of the target such as position, velocity, and probably trajectory [6]. Bayesian filtering uses recurrent modeling for better state approximation outcomes of real-time nonlinear phenomena [7,8].

In particular, bearings-only passive target tracking is a typical nonlinear filtering problem that is usually related to target motion analysis (TMA) [9]. In Bearings-only tracking (BOT), the major goal is to efficiently estimate the dynamics of the single or multi moving targets by means of noise-corrupted passive measurements from array elements localized on observation platforms [10]. In recent years, BOT has gotten a lot of interest because of its significance and wide uses in a variety of practical applications like aircraft surveillance [11], underwater SONAR tracking [12], navigation [13], passive target tracking [14], etc., in which a real-time state of object is found by using only the bearings’ passive measurements.

In general, following the Bayesian filtering procedure, both predicted movement of the concerned object and probability of the collected passive measurements are used to compute the posterior likelihood density of the object kinetics [15]. After that an accurate estimate of the target dynamics is calculated from this posterior function. However, due to a large number of nonlinearities and ambiguities in the measurement model of the BOT problem, reliable state estimation results from the Bayesian approach is still a challenging research task [16]. By using the Bayesian methodology, researchers have developed many nonlinear filters to handle state estimation problem in an iterative pattern, like extended Kalman filter (EKF) [17], unscented Kalman filter (UKF) [18,19], Gauss–Hermite quadrature filter (GHQF) [20], cubature Kalman filter (CKF) [21,22,23], Gaussian filter [24], sparse grid quadrature filter (SGQF) [25], and a Gaussian sum based cubature Kalman filter [26].

Initial sub optimum techniques, following extended Kalman filter (EKF), provide poor resolution, unstable course accuracy, and divergence. After that, unscented Kalman filtering follows the methodology of unscented transform and also refers to the moment-matching technique that is used widely in which sigma pints are selected conclusively to estimate subsequent probability density [17]. Gaussian filtering, which is also known as assume density filtering, established Gaussian distribution to find Gaussian weighted integrals in target tracking applications. The third degree numerical integration law is used to develop unscented transform for UKF and the spherical radial cubature rule for the basic CKF algorithm [27]. To further enhance the convergence of UKF and CKF, researchers have also proposed high degree CKF, SGQF, and square root UKF [28].

Along with Kalman filtering, famous sequential Monte-Carlo based techniques known as particle filters (PF) are also introduced, which offer better efficiency in tracking scenarios, but they demand complex mathematical computations [29]. Shifted Rayleigh filter (SRF) is also used for a complex target tracking application and shows almost similar results to the PF [30]. In [31], it is proved that particle filtering can achieve satisfactory results by following the methodology of the Kalman filter. Cubature and quadrature based continuous discrete-time filters are effectively applied for maneuvering object tracking by using discrete-time measurement function and continuous time dynamic modeling in [32]. Some researchers have also done comparative analysis of different nonlinear filters for especially passive bearings only target tracking applications for analyzing their worth in ocean environment [33,34,35].

These all discussed tracking methods are mainly applied for a single motion model of a target. In case of bearings-only maneuvering underwater target tracking, the uncertainty of the motion model directly affects the tracking performance [36]. Due to the complications and randomness of object dynamics, it is not suitable to precisely explain different maneuvering phases in a single model, which leads to a divergence between true dynamics of the target and state model [37]. To address this complex issue, researchers proposed an interactive multiple model (IMM) filter that can solve this problem efficiently. This technique maps the object motion model into a desired number of predefined model sets [38,39,40]. On the other hand, the analysis of radiated noise of underwater target can achieve more accurate target tracking in a certain extent. Some scholars have combined information entropy theory with a feature extraction algorithm to analyze characteristics of underwater targets [41,42].

Although a lot of literature is available for Bayesian filtering, meanwhile smoothing technology has also obtained sufficient interest in the past by following Wiener [43] and a Rauch–Tung–Striebel (RTS) smoother [44]. The RTS algorithm is a fixed interval post-processing smoother, which is usually designed on the estimates of its related Kalman filter [45]. The literature shows various applications of an RTS smoother in real-life practical problems like motion estimation, efficiency improvement, as well as in an unmanned air vehicle with a combination of linear and nonlinear Kalman filters [46,47,48].

**Figure 1:**
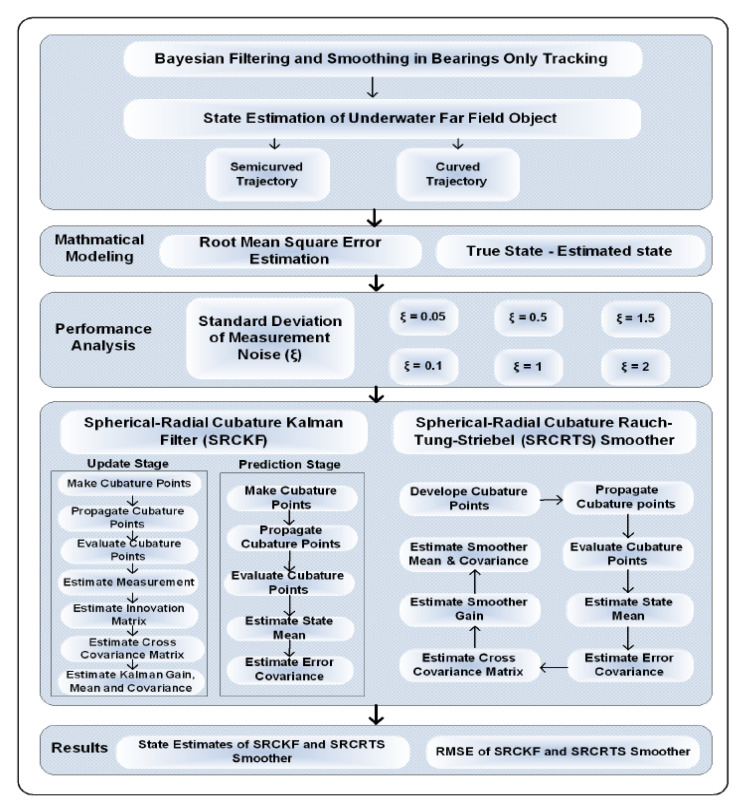
Flow chart of the Bearings Only Tracking (BOT) scheme.

Our work is inspired from all related discussion of Bayesian filtering and smoothing in target tracking applications in the context of variation in white Gaussian measurement noise for two different types of target trajectories. Both ideal and noisy clutter ocean environments are assumed in our target tracking problem by taking standard variation of measurement noise from low to high numerical values. In general, our study gives a convergence analysis of an SRCKF and RCRTS smoother in the sense of root mean square error (RMSE) between true and estimated state of targets. Here, state mean and covariance estimates of SRCKF are figured out in the forward route of both trajectories, while estimates of the SRCRTS smoother are then computed in a backward route of both trajectories. A detailed methodology of the proposed work is shown in Figure 1.

The paper is outlined in the following order. A two-dimensional Cartesian coordinates system designing is done in Section 2 for state approximation of a distant object. Mathematical equations of passive target tracking architecture are computed in this section. Bayesian filtering and smoothing algorithms are analyzed with their mathematical approach for BOT problem in Section 3. A RMSE based fitness function is evaluated for all algorithms in the form of figures and tables in Section 4. State estimates and position error results are simulated in MATLAB; in addition, convergence analysis of Kalman filtering is explained in this section. The final section of this paper reports significant contributions of the proposed methodology.

## 2. Passive Target Tracking System Model

**Figure 2:**
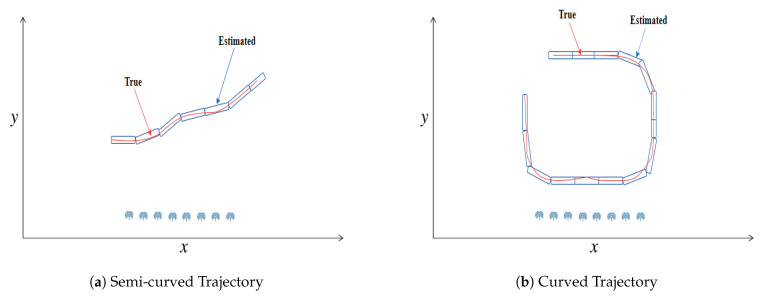
Passive target tracking framework.

A two-dimensional state-space system model for bearings-only target tracking scenario is designed in this section. The problem is based on Cartesian coordinates for efficient state estimation of a far-field moving object in the ocean environment. A Horizontal Uniform Linear Array (HULA) of eight sensors is installed on the base station for tracking purpose of the object. The passive measurements from antenna elements are bearings only of the moving object, which relies on the direction and angle of each array element. In this proposed passive target tracking model, target movement is assumed in semi-curved and curved trajectories, which we want to track with SRCKF and SRCRTS smoothers. This passive target tracking architecture is shown in Figure 2.

The state of a target is based on two-dimensional position and velocity vector at time i, which are $\left(x_{i},y_{i}\right)$ and $\left(Dx_{i},Dy_{i}\right)$, correspondingly. These parameters are defined in the state vector $A_{{}_{i}}^{\tau }$ as:

(1) $$A_{{}_{i}}^{\tau }={\left[\begin{array}{cccc} x_{{}_{i}}^{\tau } & y_{{}_{i}}^{\tau } & Dx_{{}_{i}}^{\tau } & Dy_{{}_{i}}^{\tau } \end{array}\right]}^{T}.$$

In the above state vector, ${\left[.\right]}^{T}$ denotes transpose of matrix. Similarly, the state vector of the observer on base station is defined as:

(2) $$A_{{}_{i}}^{o}={\left[\begin{array}{cccc} x_{{}_{i}}^{o} & y_{{}_{i}}^{o} & Dx_{{}_{i}}^{o} & Dy_{{}_{i}}^{o} \end{array}\right]}^{T}.$$

A relative or comparative state vector can be designed as:

(3) $$A_{i}=A_{i}^{\tau }-A_{i}^{o}={\left[\begin{array}{cccc} x_{i} & y_{i} & Dx_{i} & Dy_{i} \end{array}\right]}^{T}.$$

Assuming a nearly discrete time linear continuous Wiener velocity motion model [49] for dynamics of the moving object. The dynamic model representing state equation can be written as:

(4) $$A_{i}=\alpha_{i-1}A_{i-1}+\eta_{i-1}.$$

In the above model, $\alpha_{i-1}$ is a state transition matrix having dimensions of m×m, which describes the behavior of the dynamic model. $\eta_{i-1}$ is independent and identically distributed (IID) white Gaussian process noise, which has zero mean. Both transition matrix and process noise are given for sampling interval [$\tau_{i-1}$,$\tau_{i}$].

(5) $$\Delta \tau =\left[\tau_{i-1}-\tau_{i}\right],$$

(6) $$\alpha_{i-1}=\left[\begin{array}{cccc} 1 & 0 & \Delta \tau & 0\\ 0 & 1 & 0 & \Delta \tau \\ 0 & 0 & 1 & 0\\ 0 & 0 & 0 & 1 \end{array}\right].$$

To efficiently estimate this model of target with discrete-time Bayesian filtering and smoothing algorithms, the overall dynamic model defined above must be discretized. This discrete-time state equation is used for integrating the model exactly over sampling intervals, which are multiples of Δτ. Dynamics of the target presented in discrete time with sampling interval Δτ as:

(7) $$A_{i}=\underset{\alpha_{i-1}}{\underset{︸}{\left[\begin{array}{cccc} 1 & 0 & \Delta \tau & 0\\ 0 & 1 & 0 & \Delta \tau \\ 0 & 0 & 1 & 0\\ 0 & 0 & 0 & 1 \end{array}\right]}}\underset{A_{i-1}}{\underset{︸}{\left[\begin{array}{c} x_{i-1}\\ y_{i-1}\\ Dx_{i-1}\\ Dy_{i-1} \end{array}\right]}}+\eta_{i-1}.$$

The covariance of white Gaussian process noise is defined by $\varphi_{i-1}$ as:

(8) $$\eta_{i-1}∼N(0,\varphi_{i-1}),$$

(9) $$\varphi_{i-1}=E\left[{\eta_{i-1}\eta_{i-1}}^{T}\right].$$

Meanwhile, process noise in the dynamic model should be discretized to obtain a discrete-time state equation, which is also necessary for integrating the dynamic model accurately over sampling intervals. The above equation is updated in the form of a covariance matrix as:

(10) $$\varphi_{i-1}=\left[\begin{array}{cccc} \frac{1}{3}\Delta \tau^{3} & 0 & \frac{1}{2}\Delta \tau^{2} & 0\\ 0 & \frac{1}{3}\Delta \tau^{3} & 0 & \frac{1}{2}\Delta \tau^{2}\\ \frac{1}{2}\Delta \tau^{2} & 0 & \Delta \tau & 0\\ 0 & \frac{1}{2}\Delta \tau^{2} & 0 & \Delta \tau \end{array}\right]\omega ,$$

where scalar ω is the spectral intensity of the process noise. Furthermore, the measurement equation is also referred to as the state-space model. The measurement model at time step i is described as:

(11) $$B_{i}=\beta \left(A_{i},\nu_{i}\right).$$

Measurement function β(.) in the above model is based on current measurements at time i. It consists of noise-deprived bearings from the base station platform to the object, while ν is denoting independent Gaussian measurement noise. The relationship of the real-time position of the object and measured bearings is described in measurement model B for sensor j at time step i as:

(12) $$B_{i}^{j}=\arctan\left[\frac{y_{i}-\lambda_{y}^{j}}{x_{i}-\lambda_{x}^{j}}\right]+\nu_{i}^{j}.$$

The orientation of sensors j in Cartesian coordinates is denoted by ($\lambda_{y}^{j}$,$\lambda_{x}^{j}$) in the above measurement model, whereas independent white Gaussian measurement noise is $\nu_{i}^{j}$, which has zero mean with covariance X.

(13) $$\nu_{i}^{j}∼N\left(0,X\right),$$

(14) $$X=\mathrm{diag}(\xi_{B}^{2}).$$

In Equation (14), a standard deviation of measurement noise is represented by $\xi_{B}$ and measured in radian, which is a key parameter in the passive target framework. In our work, we varied this standard deviation of measurement noise ξ for checking the convergence and performance of Bayesian filtering and smoothing algorithms over a fixed number of array elements j. Assuming that the initial state of moving object is $A_{0}$ = ${\left[-2\;-0.5\;1\;0\right]}^{T}$ and, in state estimation of the moving target, previous distribution for the starting state is $A_{0}$∼ N(0, $M_{0}$), while $M_{0}$ is shown as:

(15) $$M_{0}=\left[\begin{array}{cccc} 0.1 & 0 & 0 & 0\\ 0 & 0.1 & 0 & 0\\ 0 & 0 & 10 & 0\\ 0 & 0 & 0 & 10 \end{array}\right].$$

From the above prior distribution matrix, it is obvious that target’s position is of more interest in this study than the target’s velocity. For getting semi-curved and curved trajectory in simulations, a slight random acceleration is given to an object.

## 3. Bayesian Filtering and Smoothing Algorithms

This section explains the methodology of both Bayesian filtering and smoothing algorithms SRCKF and SRCRTS smoother in detail with their mathematical expressions.

### 3.1. Spherical-Radial Cubature Kalman Filter

Spherical Radial Cubature Kalman filter (SRCKF) is based on the principle of well known spherical–radial cubature transformation, which exploits the strength of assumed density phenomena. Spherical–radial cubature transform numerically estimates the multidimensional integrals used in Bayesian filtering by creating cubature points with normalized weights. In this phenomenon, the numerical integration technique is applied differently from Gauss–Hermite transformation, which uses product rule. SRCKF is considered a more stable nonlinear filtering algorithm according to the numerical stability factor with better numerical characteristics. It is numerically accurate and can provide an efficient solution for complex nonlinear filtering problems. SRCKF is an iterative optimal state approximation technique that is widely applied for measurement and designing complexities. In SRCKF, initially an array of cubature points is molded, which are correlated with equivalent weights. These cubature points are then circulated throughout the dynamic and measurement equations of the state-space model. In the workflow of the filter, state mean and state covariance are calculated from past observations following time updates. In the next phase, the gain of the filter is computed and then correlations are applied by following measurement updates. Different methodology steps involved in the function of SRCKF, which move recursively are described here. Let us consider the subsequent density function $h\left(a_{i-1}|b_{i-1}\right)=N(y_{i-1|i-1},z_{i-1|i-1})$ with state mean y and state covariance z is known at time step i = 1,…,Δτ.

- Prediction Phase:
  1. Pick cubature points $\chi_{k}$, while k = 1,…,2p from the interchange of the p size unit sphere and Cartesian coordinates. Adjust these points by $\sqrt{p}$ shown as:
    (16) $$\chi_{k}=\left\{\begin{array}{c} \sqrt{p}c_{k}\;\;\;\;\;,k=1,\ldots ,p,\\ -\sqrt{p}c_{k-p}\;,k=p+1,\ldots ,2p. \end{array}\right.$$
  2. Transmit the cubature points in a state-space dynamic model. The lower triangular Cholesky factor is shown in a square root matrix as:
    (17) $$A_{k,i-1|i-1}=\sqrt{z_{i-1|i-1}}\chi_{k}+y_{i-1|i-1}.$$
  3. Then, assess the cubature points with the dynamic model function as:
    (18) $$A_{k,i|i-1}^{*}=\alpha A_{k,i-1|i-1}.$$
  4. The predicted state mean is computed as:
    (19) $$y_{i|i-1}=\frac{1}{2p}\sum_{k=1}^{2p}A_{k,i|i-1}^{*}.$$
  5. The predicted error covariance is calculated as:
    (20) $$z_{i|i-1}=\frac{1}{2p}\sum_{k=1}^{2p}A_{k,i|i-1}^{*}A_{k,i|i-1}^{*T}-y_{i|i-1}{y_{i|i-1}}^{T}+\varphi_{i-1}.$$
- Update Phase:
  1. In the first step of the measurement update phase, again develop cubature points $\chi_{k}$, where k = 1,…,2p from the relationship of the p length unit sphere and the *x*–*y* axes. Calibrate them by $\sqrt{p}$.
  2. Circulate the cubature points in state equation of dynamic model as:
    (21) $$A_{k,i|i-1}=\sqrt{z_{i|i-1}}\chi_{k}+y_{i|i-1}.$$
  3. Classify these cubature points owing to the state equation of the measurement model function as:
    (22) $$B_{k,i|i-1}=\beta \left(A_{k,i|i-1}\right).$$
  4. Predicted measurement is approximated as:
    (23) $${\hat{b}}_{i|i-1}=\frac{1}{2p}\sum_{k=1}^{2p}B_{k,i|i-1}.$$
  5. Then, the innovation covariance matrix is computed as:
    (24) $$\Psi_{i|i-1}=\frac{1}{2p}\sum_{k=1}^{2p}B_{k,i|i-1}{B_{k,i|i-1}}^{T}-{{\hat{b}}_{k,i|i-1}{\hat{b}}_{k,i|i-1}}^{T}+X_{i}.$$
  6. The cross-covariance matrix is estimated as:
    (25) $$z_{ab,i|i-1}=\frac{1}{2p}\sum_{k=1}^{2p}A_{k,i|i-1}{B_{k,i|i-1}}^{T}-{y_{i|i-1}{\hat{b}}_{i|i-1}}^{T}.$$
  7. In the final step gain of the filter, state mean and state covariance terms are calculated as:
    (26) $$Q_{i}=z_{ab,i|i-1}\Psi_{i|i-1}^{-1},$$
    (27) $$y_{i|i}=y_{i|i-1}+Q_{i}\left(b_{i}-{\hat{b}}_{i}\right),$$
    (28) $$z_{i|i}=z_{i|i-1}-Q_{i}z_{bb,i|i-1}{Q_{i}}^{T}.$$

### 3.2. Spherical-Radial Cubature Rauch–Tung–Striebel Smoother

In this part of the study, the methodology of a Spherical-Radial Cubature Rauch–Tung–Striebel (SRCRTS) smoother is discussed for efficient state estimation of a far-field moving target. It is noticeable that the smoother is always applied to the available estimates of its corresponding filter. In other words, the smoother is a post-processing technique for acquiring more refined estimates. Basically, a smoother is an optimal estimation algorithm that responds on time instant Δτ and utilizes those measurements that are completed after time instant Δτ. Accuracy and convergence of smoother are always greater than a typical filter because it uses additional values in the form of passive measurements for its operation than filter. Here, mathematical modeling of SRCRTS is designed over cubature filtering for bearing only passive target tracking structure. We assume that the state mean of filter $y_{k|k}$ and state covariance of filter $z_{k|k}$ are established jointly with smoothing outcome $h\left(a_{i+1}|b_{1:\Delta \tau }\right)=N(y_{i+1|\Delta \tau },z_{i+1|\Delta \tau })$.

1. Make cubature points $\chi_{k}$, while k = 1,…,2p from the junction of the p size unit sphere and the Cartesian coordinates. Regulate cubature points by $\sqrt{p}$ shown as:
  (29) $$\chi_{k}=\left\{\begin{array}{c} \sqrt{p}c_{k}\;\;\;\;\;,k=1,\ldots ,p,\\ -\sqrt{p}c_{k-p}\;,k=p+1,\ldots ,2p. \end{array}\right.$$
2. Cubature points are circulated in state space equation as:
  (30) $$A_{k,i|i}=\sqrt{z_{i|i}}\chi_{k}+y_{i|i}.$$
3. Then, cubature points are checked with the dynamic model function as:
  (31) $$A_{k,i+1|i}^{*}=\alpha A_{k,i|i}.$$
4. Predict state mean as:
  (32) $$y_{i+1|i}=\frac{1}{2p}\sum_{k=1}^{2p}A_{k,i+1|i}^{*}.$$
5. Predict error covariance as:
  (33) $$z_{i+1|i}=\frac{1}{2p}\sum_{k=1}^{2p}{A_{k,i+1|i}^{*}A_{k,i+1|i}^{*}}^{T}-y_{i+1|i}{y_{i+1|i}}^{T}+\varphi_{i}.$$
6. Cross-covariance matrix is computed as:
  (34) $$\Gamma_{i,i+1}=\frac{1}{2p}\sum_{k=1}^{2p}\left(A_{k,i|i}-y_{i|i}\right){\left(A_{k,i+1|i}^{*}-y_{i+1|i}\right)}^{T}.$$
7. Smoother gain $R_{i}$ is computed together with the smoother state mean and covariance as:
  (35) $$R_{i}=\Gamma_{i,i+1}z_{i+1|i}^{-1},$$
  (36) $$y_{i|\Delta \tau }=y_{i|i}+R_{i}\left(y_{i+1|\Delta \tau }-y_{i+1|i}\right),$$
  (37) $$z_{i|\Delta \tau }=z_{i|i}+R_{i}\left(z_{i+1|\Delta \tau }-z_{i+1|i}\right){R_{i}}^{T}.$$

The convergence and effectiveness of Bayesian filtering and smoothing techniques are calculated in the sense of least position difference among actual and estimated position of dynamic targets at each time instant Δτ in meters. Root Mean Square Error (RMSE) ε of SRCKF, SRCRTS, UKF, and URTS is computed for every Monte Carlo simulation as:

(38) $$\varepsilon \left(\Delta \tau \right)=\sqrt{\frac{1}{N_{t}}\sum_{t=1}^{N_{t}}{∥A_{\Delta \tau }^{True}-A_{\Delta \tau }^{Est}∥}^{2}}.$$

In the above fitness evaluation function, the total number of independent Monte Carlo runs are denoted with $N_{t}$, estimated state of target is $A_{\Delta \tau }^{Est}$ and true state is represented by $A_{\Delta \tau }^{True}$ for t Monte Carlo simulations at time instant Δτ.

## 4. Simulation and Results

Simulation results in the form of state estimates and position errors of SRCKF, SRCRTS, UKF, and URTS are discussed briefly in this section. The performance of both methods is analyzed with respect to variation in the standard deviation of measurement noise from 0.05 to 2 radian. Different parameters of a passive target tracking framework used in Monte Carlo simulations are given in Table 1 with their appropriate values.

Ocean medium is always considered a complicated environment for target tracking applications. In particular, measurement noise is totally ambiguous in its nature. We consider two different trajectories for checking the performance of both techniques; for this, we made two cases for efficient presentation of simulation results. In both cases, we vary standard variation of measurement noise from 0.05 to 2 radian for analyzing the effectiveness of both algorithms. These two cases are briefly explained here with their simulation results in the form of figures and tables.

### 4.1. State Estimation of Semi-Curved Trajectory with Respect to Standard Deviation of Measurement Noise

**Figure 3:**
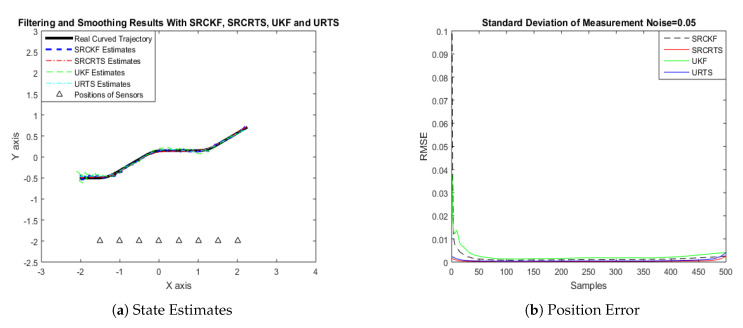
Tracking performance of Spherical Radial Cubature Kalman Filter (SRCKF), Spherical Radial Cubature Rauch–Tung–Striebel (SRCRTS), Unscented Kalman Filter (UKF), and Unscented Rauch–Tung–Striebel (URTS) for measurement noise of 0.05 rad.

**Figure 4:**
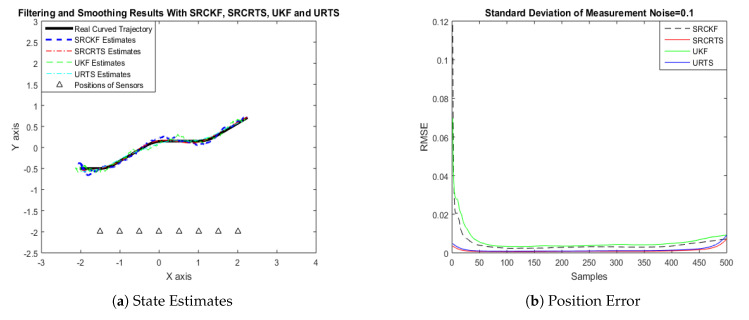
Tracking performance of SRCKF, SRCRTS, UKF, and URTS for measurement noise of 0.1 rad.

**Figure 5:**
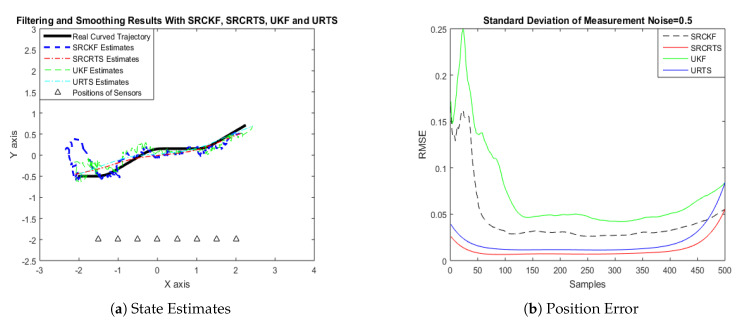
Tracking performance of SRCKF, SRCRTS, UKF, and URTS for measurement noise of 0.5 rad.

**Figure 6:**
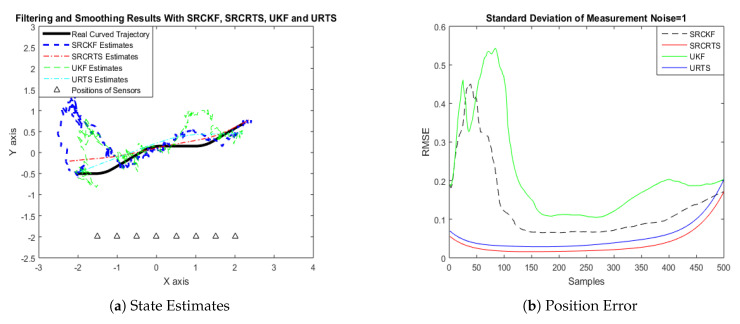
Tracking performance of SRCKF, SRCRTS, UKF, and URTS for measurement noise of 1 rad.

**Figure 7:**
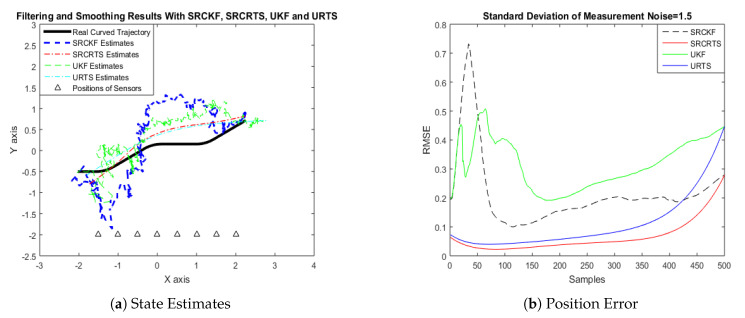
Tracking performance of SRCKF, SRCRTS, UKF, and URTS for measurement noise of 1.5 rad.

**Figure 8:**
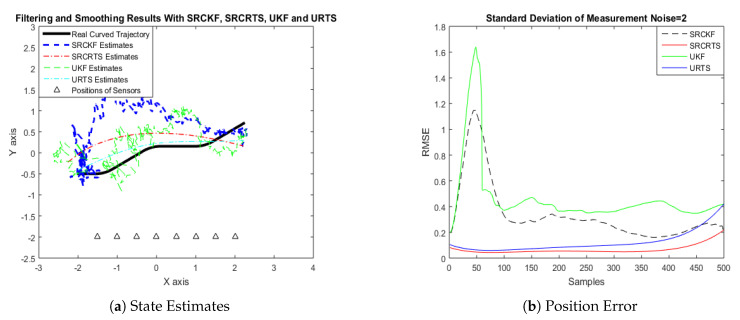
Tracking performance of SRCKF, SRCRTS, UKF, and URTS for measurement noise of 2 rad.

In this case of simulation results, accuracy and convergence of SRCKF and SRCRTS smoothers are analyzed with respect to UKF and URTS for tracking the semi-curved trajectory of a moving object. The standard deviation of measurement noise fluctuates from 0.05 to 2 radian over 500 independent Monte Carlo simulations. At every real-time instant, state estimation of targets is observed with eight array elements that are installed on a horizontal linear array at the base station. The location of array elements in Cartesian coordinates is starting from (−1.5,−2) and, by keeoing *y*-axis steady, the last sensor is localized on (2,−2). In mathematical modeling, the position of sensors is represented by $\lambda_{y}^{j}$,$\lambda_{x}^{j}$ in measurement model B. In Equations (12) to (14), the measurement model is updating for every variation in standard deviation measurement noise ξ. Effectiveness of SRCKF and SRCRTS smoothers for sufficient numerical values of standard deviation of measurement noise is shown here in the pattern of state estimates and least position error in meters from Figure 3, Figure 4, Figure 5, Figure 6, Figure 7 and Figure 8.

It is obvious from the above results that, when we increase the standard deviation of measured noise, initially SRCKF practices several difficulties for detecting a real semi-curved trajectory. The reason for this phenomenon is due to comparatively large ambiguity in the starting velocity, which can be observed in all simulation results. Convergence and accuracy of SRCKF are getting better with increasing the samples; this is due to the fact that every estimation technique has the capability to correct its convergence according to time and samples. From a comparative point of view, it is worth noticing that the performance of SRCKF and SRCRTS smoothers is far better from UKF and URTS in all scenarios of measured noise. In addition, smoothers show a better convergence rate than their corresponding filters. The reason is simple that the smoother uses more observations for its estimates than the filter, which is already discussed in the previous section.

In noisy ocean environments, where standard deviation of measurement noise is high, it is observed that, before one-third of the total samples, the convergence of SRCKF is bad. After half of the total samples, the convergence rate of SRCKF is getting better and at the end of the trajectory; it is almost approaching smoother results. The above results for a semi-curved trajectory indicate that, when we take higher values of measurement noise, the convergence of both techniques is declining exponentially and, in results, the position error is increasing. However, comparatively, convergence of spherical–radial cubature transformation is better in all scenarios from unscented transformation. The mean of position errors that is measured in meters in the form of Root Mean Square Error (RMSE) is listed here in Table 2. RMSE is an evaluation function for checking the convergence of both techniques in this study. These position errors are computed for both algorithms with respect to each variation of measured noise for 500 independent samples. Results indicated in Table 2 also endorse the previous results that are described in the above figures. It can be seen that the accuracy of smoothers is almost double the number of filters in all scenarios, which is clearly evident for the effectiveness of the smoothing algorithm.

### 4.2. State Estimation of a Curved Trajectory with Respect to the Standard Deviation of Measurement Noise

Here, in this case, convergence analysis of all algorithms is done for a curved trajectory of the far-field moving target. The standard deviation of measurement noise is also varied in this case from 0.05 to 2 radian for checking the performance of SRCKF, SRCRTS, UKF, and URTS over 500 independent Monte Carlo samples. In this case, there are also eight sensors for getting the passive measurements from a far-field moving object that is roaming in the curved trajectory. Like the previous case, these sensors are also localized on a horizontal uniform linear array at the base station.

**Figure 9:**
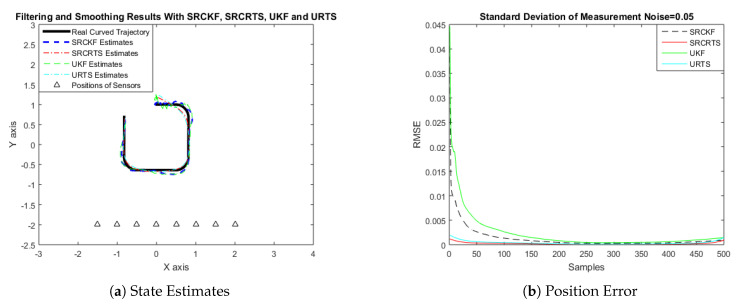
Tracking performance of SRCKF, SRCRTS, UKF, and URTS for measurement noise of 0.05 rad.

**Figure 10:**
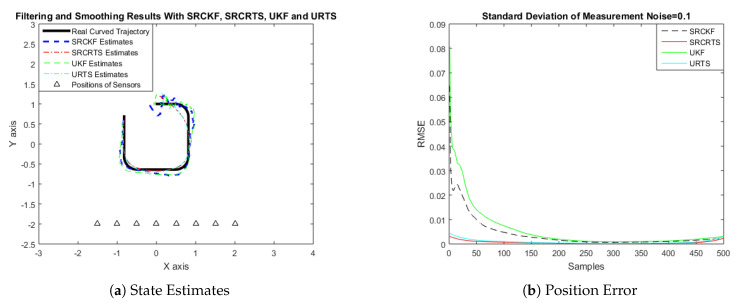
Tracking performance of SRCKF, SRCRTS, UKF, and URTS for measurement noise of 0.1 rad.

**Figure 11:**
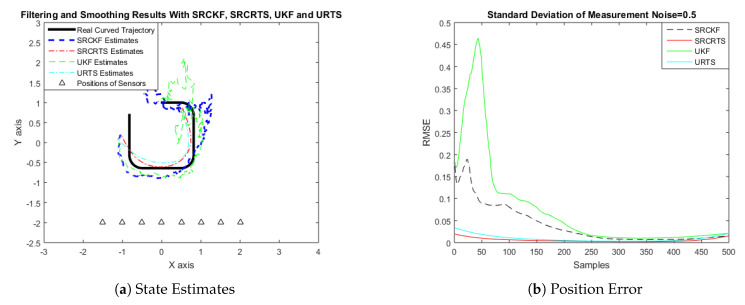
Tracking performance of SRCKF, SRCRTS, UKF, and URTS for measurement noise of 0.5 rad.

**Figure 12:**
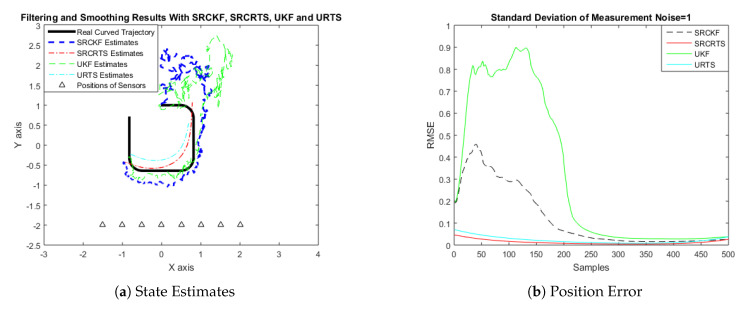
Tracking performance of SRCKF, SRCRTS, UKF, and URTS for measurement noise of 1 rad.

**Figure 13:**
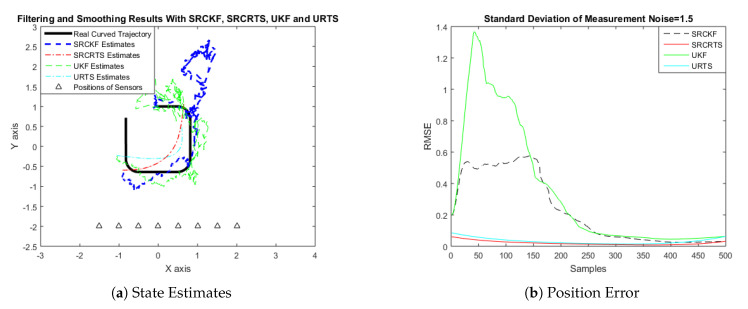
Tracking performance of SRCKF, SRCRTS, UKF, and URTS for measurement noise of 1.5 rad.

**Figure 14:**
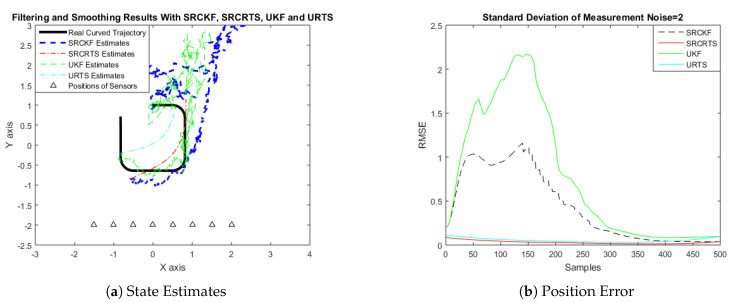
Tracking performance of SRCKF, SRCRTS, UKF, and URTS for measurement noise of 2 rad.

In the below results, efficient target tracking is done for the curved trajectory in the form of state estimates and position errors. The performance of SRCKF and SRCRTS is analyzed in a comparison of UKF and URTS for 500 samples. The trend of both algorithms is almost identical as discussed in the previous case. Accuracy and convergence for the curved trajectory are slightly less from a semi-curved trajectory, which is noticeable because of more acceleration given in simulations for the curved trajectory. Again, like the previous case, for all variations of measurement noise from 0.05 to 2 radian, better results are observed from smoothers in comparison of filters. Bayesian filtering and smoothing results in the pattern of state estimates of curved trajectory and position error are shown from Figure 9, Figure 10, Figure 11, Figure 12, Figure 13 and Figure 14.

The argument of better performance of SRCRTS and URTS is the same as discussed previously—that the smoother is using more observation than the filter for tracking the curved trajectory of the target. In this case, SRCKF is also experiencing many troubles at the start of the trajectory to find the accurate route because of uncertainties in initial velocities. In ideal ocean environments, where standard deviation of measurement noise is low, the convergence rate of SRCKF filter is at a minimum at the start and the middle of the trajectory, but, after that, SRCKF is gradually improving its performance. For example, at ξ = 0.1 radian, the filter is converging around about 300 samples and, after that, it is showing good performance until the last sample. It is clear from all given results that accuracy of both SRCKF and UKF is exponentially declining with increasing numerical values of measured noise due to cluttered and noisy ocean environments. SRCRTS is also showing better tracking capability in this case with respect to URTS even in noisy conditions. In the last portion of the trajectory, tracking capability of both algorithms is nearly identical. In this case, position error results are also shown in Table 3 over 500 samples. These observations also validate previous results reported in case 1 that cubature filtering is showing better performance even for curved trajectory for all scenarios of measured noise. Position errors of both algorithms are linearly increasing with variation in values of measured noise, which is shown in Table 3.

## 5. Conclusions

Strength of nonlinear Bayesian filtering through SRCKF, UKF algorithms, and smoothing based on SRCRTS and URTS algorithms is efficiently and effectively exploited for bearings only passive target tracking problem arises in ocean environment studies. In this problem, moving target is assumed to be in the far field and a recently reported state estimation of the target at every time instant is analyzed in two-dimensional Cartesian coordinate systems. Initially, a state space based dynamic and measurement models are mathematically designed by using the phenomena of BOT. After that, the proposed methodology of both SRCKF and SRCRTS methods is exploited for a given problem of passive target tracking. These Bayesian filtering and smoothing algorithms are simulated for 500 independent samples. Convergence and accuracy of all algorithms are examined for two scenarios based on semi-curved and curved trajectory of target motion and measure the performance on RMSE metrics. In each case study, different variations of white Gaussian measurement noise are applied to establish the robust and reliable behavior of proposed algorithms. All simulation results clearly demonstrate that performance of the smoothing algorithm is far better from a filtering technique. A trade-off is observed among computational complexity and accuracy of SRCRTS and URTS smoothers. The rise in standard deviation of measurement noise results in exponential decay in the accuracy of both algorithms for each case of the passive tracking problem and vice versa. In future, one may investigate the fractional adaptive filtering algorithms [50,51,52,53] for achieving better state estimation results in an underwater noisy medium, which is still a challenging research domain and has a wide capability for progress and expansion.

## Figures and Tables

**Table 1:** Settings of parameters for a passive target tracking scheme.

Parameters	Values
Initial location of target in coordinates	$A_{0}$ = ${\left[-2\;-0.5\;1\;0\right]}^{T}$
Location of array elements	($\lambda_{-1.5\to 2}^{j}$,$\lambda_{-2}^{j}$)
Number of antennas	j = 8
Spectral intensity of process noise	ω = 0.1
Covariance of measurement noise	z = $\mathrm{diag}(\xi_{B}^{2})$ ξ = 0.05→2 rad
Initial state covariance of cubature filter	z = diag([0.1 0.1 … 10])
Time duration	dt = 0.01
Number of steps for target trajectory	1
Number of samples	500
Space between array elements	0.5

**Table 2:** Root mean square errors of filtering and smoothing algorithms by varying standard deviation of measurement noise for a semi-curved trajectory.

Noise (rad)	SRCKF RMSE (m)	SRCRTS RMSE (m)	UKF RMSE (m)	URTS RMSE (m)
ξ = 0.05	0.0410	0.0195	0.0510	0.0255
ξ = 0.1	0.0675	0.0334	0.0762	0.0388
ξ= 0.5	0.2084	0.1063	0.2689	0.1367
ξ = 1	0.3661	0.1897	0.4632	0.2296
ξ = 1.5	0.4645	0.2531	0.5632	0.3290
ξ = 2	0.6254	0.2717	0.6841	0.3478

**Table 3:** Root mean square errors of filtering and smoothing algorithms by varying standard deviation of measurement noise for curved trajectory.

Noise (rad)	SRCKF RMSE (m)	SRCRTS RMSE (m)	UKF RMSE (m)	URTS RMSE (m)
ξ = 0.05	0.0351	0.0146	0.0479	0.0191
ξ = 0.1	0.0625	0.0249	0.0752	0.0292
ξ = 0.5	0.1984	0.0743	0.2756	0.0948
ξ = 1	0.3487	0.1138	0.5601	0.1488
ξ = 1.5	0.4776	0.1436	0.5912	0.1798
ξ = 2	0.6640	0.1755	0.8835	0.2318
